# Overview of BioCreative II gene normalization

**DOI:** 10.1186/gb-2008-9-s2-s3

**Published:** 2008-09-01

**Authors:** Alexander A Morgan, Zhiyong Lu, Xinglong Wang, Aaron M Cohen, Juliane Fluck, Patrick Ruch, Anna Divoli, Katrin Fundel, Robert Leaman, Jörg Hakenberg, Chengjie Sun, Heng-hui Liu, Rafael Torres, Michael Krauthammer, William W Lau, Hongfang Liu, Chun-Nan Hsu, Martijn Schuemie, K Bretonnel Cohen, Lynette Hirschman

**Affiliations:** 1 Biomedical Informatics, Stanford University, 251 Campus Drive,, Stanford, CA, 94305, USA; 2 Center for Computational Pharmacology, University of Colorado School of Medicine, PO Box 6511, Aurora, Colorado, 80045, USA; 3 School of Informatics, University of Edinburgh, 2 Buccleuch Place, Edinburgh, EH8 9LW, UK; 4 Oregon Health & Science University, 3181 SW Sam Jackson Park Road, Portland, Oregon, 97239, USA; 5 Fraunhofer Institute for Algorithms and Scientific Computing (SCAI), Schloss Birlinghoven, D-53754, Sankt Augustin, Germany; 6 University and Hospitals of Geneva, 24 Micheli du Crest, 1201 Geneva, Switzerland; 7 School of Information, University of California, Berkeley, 102 South Hall, Berkeley, California, 94720, USA; 8 Institut für Informatik, Ludwig-Maximilians-Universität München, Amalienstr. 17, 80333 Munich, Germany; 9 Department of Computer Science and Engineering, Arizona State University, 699 S. Mill Avenue, Tempe, Arizona, 85281, USA; 10 Biotechnological Centre, Technische Universität Dresden, Tatzberg 47-51, 1307 Dresden, Germany; 11 School of Computer Science and Technology, Harbin Institute of Technology, West Da-Zhi Street, Mailbox 319, No. 92, Harbin, 150001, China; 12 Department of Computer Science and Information Engineering, National Cheng Kung University, No. 1, University Road, Tainan City 701, Taiwan; 13 Bioalma, Biolma, Ronda de Poniente, 4, 2 C-D, Tres Cantos, Madrid, E-28760, Spain; 14 Department of Pathology, Yale University School of Medicine, 300 Cedar Street TAC 309, New Haven, Connecticut, 06510, USA; 15 Division of Computational Bioscience, Center for Information Technology, National Institutes of Health, 9000 Rockville Pike, Bethesda, Maryland, 20892, USA; 16 Department of Biostatistics, Bioinformatics and Biomathematics, Georgetown University Medical Center, 4000 Reservoir Rd. NW, Washington, District of Columbia, 20057, USA; 17 Institute of Information Science, Academia Sinica, 128 Academic Road, Section 2, Taipei, Taiwan; 18 Biosemantics Group, Medical Informatics Department, Erasmus MC University Medical Center, Dr. Molewaterplein 50, 3015GE, Rotterdam, The Netherlands; 19 Information Technology Center, The MITRE Corporation, 202 Burlington Road, Bedford, Massachusetts, 01730 USA

## Abstract

**Background:**

The goal of the gene normalization task is to link genes or gene products mentioned in the literature to biological databases. This is a key step in an accurate search of the biological literature. It is a challenging task, even for the human expert; genes are often described rather than referred to by gene symbol and, confusingly, one gene name may refer to different genes (often from different organisms). For BioCreative II, the task was to list the Entrez Gene identifiers for human genes or gene products mentioned in PubMed/MEDLINE abstracts. We selected abstracts associated with articles previously curated for human genes. We provided 281 expert-annotated abstracts containing 684 gene identifiers for training, and a blind test set of 262 documents containing 785 identifiers, with a gold standard created by expert annotators. Inter-annotator agreement was measured at over 90%.

**Results:**

Twenty groups submitted one to three runs each, for a total of 54 runs. Three systems achieved F-measures (balanced precision and recall) between 0.80 and 0.81. Combining the system outputs using simple voting schemes and classifiers obtained improved results; the best composite system achieved an F-measure of 0.92 with 10-fold cross-validation. A 'maximum recall' system based on the pooled responses of all participants gave a recall of 0.97 (with precision 0.23), identifying 763 out of 785 identifiers.

**Conclusion:**

Major advances for the BioCreative II gene normalization task include broader participation (20 versus 8 teams) and a pooled system performance comparable to human experts, at over 90% agreement. These results show promise as tools to link the literature with biological databases.

## Background

**Figure 1:**
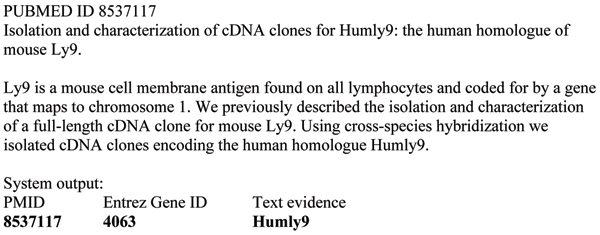
Sample PubMed/MEDLINE abstract and extracted Entrez Gene identifiers.

The goal of the gene normalization (GN) task is to determine the unique identifiers of genes and proteins mentioned in scientific literature. For the BioCreative II GN task, the identifiers are Entrez Gene IDs, the genes and proteins are associated with humans, and the targeted literature is a collection of abstracts taken from PubMed/MEDLINE. Gene normalization is a challenging task even for the human expert; despite the existence of various standards bodies, there is great variability in how genes and gene products are mentioned in the literature. There are two problems. First, genes are often described, rather than referred to by gene name or symbol, as in 'p65 subunit of NF-kappaB' or 'light chain-3 of microtubule-associated proteins 1A and 1B.' This can make correct association with the Entrez Gene identifier difficult. Second, gene mentions can be ambiguous. Figure 1 shows a sample PubMed/MEDLINE abstract that discusses the gene 'Humly9'. However, a search on 'Humly9' in Entrez Gene returns no hits; a search on 'Ly9' returns hits from multiple organisms (mouse, human, dog, and so on) as well as from multiple genes, including Slamf7 (mouse), which has 'novel Ly9' as a synonym, and SLAMF7 (human, identifier 57823), with synonym 'novel LY9 (lymphocyte antigen 9) like protein'.

This example illustrates the difficulty in resolving even seemingly straightforward mentions of genes to their unique identifiers. However, the linkages that can be created when this resolution is accomplished are critical to accurate search of the literature, which contains the records of experimental evidence needed for characterization of the gene and associated gene products. The GN task was inspired by a step in a typical curation pipeline for model organism databases: once an article has been selected for curation, a curator will list the genes or proteins of interest in the article. This is a time-consuming step - both curators and researchers would benefit from tools to speed up the process of linking literature to biological databases.

The first BioCreative shared task [1,2] stimulated significant subsequent research. For example, Cohen [3] and Xu and coworkers [4] showed how existing knowledge sources could be efficiently leveraged to improve performance on the GN problem itself. Other researchers have shown the relevance of the GN task to pervasive problems in retrieving relevant documents. Sehgal and Srinivasan [5] presented a detailed analysis of the interactions between gene normalization and information retrieval, and presented an effective technique for using data from Entrez Gene summary and product information fields for solving ambiguity issues in the latter. Fang and coworkers [6] presented a detailed analysis of various approaches to the normalization of human genes, which are the target of their group's FABLE information retrieval application for human-gene-specific publications.

In the first BioCreative, the focus was on extraction of unique gene identifiers from the fly, mouse and yeast model organism databases. For BioCreative II we chose to focus on human gene and protein names, motivated in part by a desire to align more closely with the protein-protein interaction (PPI) task [7], in which a normalization step is required to map interactor proteins to UniProt IDs.

In contrast with genomic data for fly, mouse, and yeast, data for the human genome is not organized into a single model organism database. This made collection of resources somewhat more complicated. To provide a pool of articles containing information on human genes and gene products, we drew from expert-annotated data created by the Gene Ontology Annotation (GOA) team at the European Bioinformatics Institute. From this set, we provided 281 expert-annotated abstracts for training, and a blind test set of 262 documents, with a gold standard created by expert annotators; inter-annotator agreement was measured at over 90%.

As constructed, the GN task for BioCreative II represents a simplification of the real curation task. In the real process, the curator generally works from the full text of the articles, and identifies only particular kinds of genes of interest (for example, only genes for a specific organism or only genes that have experimental evidence for their function). We simplified the BioCreative GN task in three respects: we used only freely available abstracts from PubMed/MEDLINE, rather than full-text articles; we chose a set of abstracts known to be 'enriched' for human genes; and we required that every human gene or protein mentioned in the abstract be associated with an Entrez Gene identifier (and only human genes or proteins). These simplifications may help to explain discrepancies in performance on the 'idealized' GN task described here and the performance of systems on a more realistic protein normalization task performed in the context of the PPI task [7]. This latter task required normalization of proteins from full-text articles discussing multiple species - a much more challenging problem.

## Results

### Overview

We received a total of 54 runs from 20 participating teams. For each run we computed the results based on a simple matching of gene identifiers against the gold standard for each abstract. Identifiers that matched the answer key constituted true positives (TP), identifiers that did not match were false positives (FP), and gold standard identifiers that were not matched were false negatives (FN). Recall, precision, and F-measure were computed in the usual way:

• Recall = TP/(TP+FN)

• Precision = TP/(TP+FP)

• F-measure = (2*P*R)/(P+R)

We computed two sets of results: the micro-averaged results, which combined the results across identifiers in all documents to provide recall and precision scores, and the associated F-measure; and the macro-averaged results, computed on a per-document basis and then averaged across documents to obtain precision, recall and F-measure. The micro-average weights each gene identifier equally; the macro-average weights each document equally, regardless of how many genes are found in the document. Macro-averaged results provide a straightforward way to compute statistical significance. Table 1 shows the results of the top-scoring run for each team, including recall, precision, and F-measure for the best (micro-averaged) run of each system. In addition, the table shows the macro-averaged F-measure and rank, as well as the rank of the systems that had a significant difference in performance (at the 0.10 level for a one-sided *t*-test). The ranking of the top seven systems did not change between the macro-averaged scores and the micro-averaged scores. In three cases, the top-scoring run for the system changed for the macro-averaged scores.

**Table 1:** Recall, precision, F-measure and rank for best gene normalization run per team

Team/run	Recall	Precision	F-measure micro-average	Rank micro	F-measure macro-average	Rank macro	Significance range
T042_1	0.833	0.789	0.810	1	0.811	1	3-20
T034_1	0.815	0.792	0.804	2	0.782	2	8-20
T013_1	0.768	0.833	0.799	3	0.779	3	8-20
T004_1	0.734	0.841	0.784	4	**‡**0.777	4	8-20
T109_1	0.824	0.743	0.781	5	0.775	5	8-20
T104_1	0.743	0.807	0.774	6	0.773	6	9-20
T101_2	0.743	0.801	0.771	7	0.755	7	10-20
T107_1	0.740	0.784	0.761	8	**0.739**	*9	12-20
T113_2	0.761	0.752	0.756	9	**0.745**	*8	11-20
T108_3	0.749	0.726	0.737	10	0.724	10	13-20
T007_2	0.703	0.746	0.724	11	**0.694**	*12	16-20
T017_1	0.708	0.720	0.714	12	**‡0.710**	*11	15-20
T110_1	0.629	0.783	0.698	13	**0.685**	*14	16-20
T111_3	0.664	0.717	0.689	14	**0.664**	*15	17-20
T030_1	0.661	0.716	0.687	15	**0.649**	*16	17-20
T006_2	0.606	0.767	0.677	16	**‡0.686**	*13	19-20
T036_1	0.713	0.520	0.602	17	0.595	17	19-20
T014_1	0.485	0.762	0.593	18	0.584	18	20
T102_3	0.790	0.425	0.552	19	0.559	19	20
T058_2	0.415	0.375	0.394	20	0.398	20	

Columns include recall, precision, F-measure and rank for micro-averaged scores, F-measure and rank for macro-averaged scores, and significance based on macro-averaged score distributions. Bold * indicates that rank for micro-average and macro-average are different; ‡ indicates that a different run was used as the high-scoring run in macro-averaged versus micro-averaged results.

**Figure 2:**
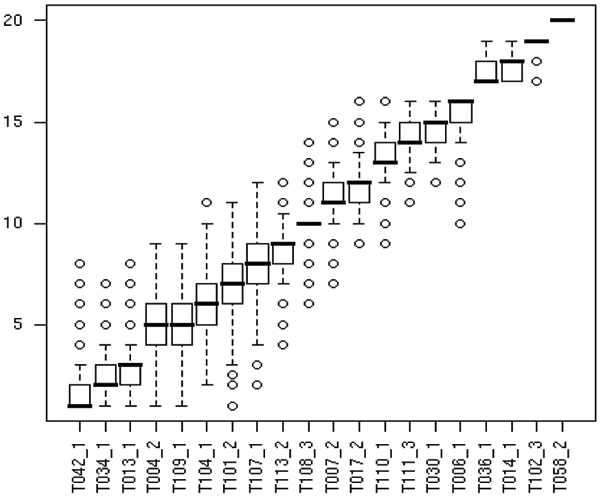
Distribution of F-measure rank computed by resampling test data over 10,000 runs. The dark line in each box plot is the median rank based on the resampling results, the boxes are quartiles around the median, the whiskers correspond roughly to the 95% confidence interval for the average rankings, and the circles are outliers.

To further evaluate the significance of the difference between system performances, we performed a bootstrap resampling analogous to that performed by Wilbur and colleagues [8]. We selected a series of 10,000 random sets of 250 PubMed/MEDLINE identifiers with replacement from the test set of 262 PubMed/MEDLINE abstracts. For each team, we chose the best-performing macro-averaged run. We then computed the micro-averaged precision, recall, and F-measure and rankings for that team, for that resampled corpus. The 10,000 resampling runs provide a distribution of rankings for each team. Figure 2 shows box plots for the distribution of F-measure rank for each submission. The dark line is the median rank, the boxes are quartiles, the whiskers correspond roughly to the 95% confidence interval for these average rankings, and the circles are outliers. From the table and the box plot, we can see the overlap in distributions. For example, team T042 is ranked first and T034 is ranked second, but there is substantial overlap in distribution; similarly, the distributions of the second- and third-ranked systems (T034 and T013) overlap, and the next two systems (T004, T109) are tied for fourth/fifth place. The top 7 systems were separated by 0.039 points of micro-averaged F-measure (0.810 to 0.771). In general, differences of less than 0.03 points of F-measure were not statistically significant at the 90% confidence level, while differences of 0.04 points or greater were significant.

**Figure 3:**
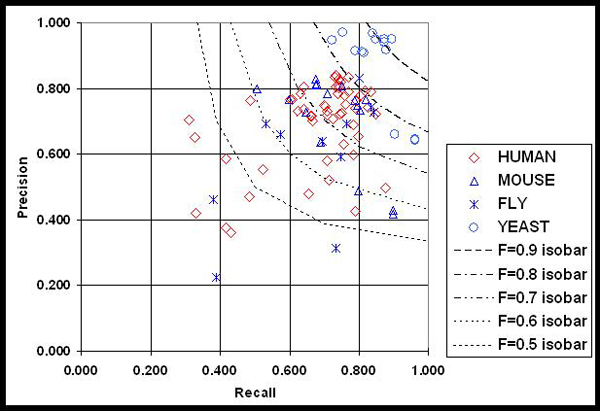
Precision versus recall scatter plot with F-measure isobars for GN micro-averaged results for human, mouse, fly and yeast data. GN, gene normalization.

Figure 3 shows the micro-averaged results as a scatter plot of precision versus recall for all 54 runs (red diamonds). The highest recall reported on an official run (T42_2) was 0.875, with a precision of 0.496, and an F-measure of 0.633. In addition, the figure shows results from the mouse, fly, and yeast results from BioCreative I (blue triangles, stars and circles) for comparison.

### Comparison with the first BioCreative challenge evaluation's GN task

One advantage of running a series of evaluations is to be able to answer the question: Is the research community making progress? To do so, it is useful to compare the results for GN in BioCreative II with the results from the first BioCreative. Table 2 shows a set of statistics for the four tasks. The top row is for human gene/proteins from BioCreative II, and the other three rows are from the first BioCreative. These statistics give some points of comparison for the complexity of the tasks. For example, yeast has a smaller genome than mouse or human, with fewer gene names, and the names are shorter than those of other species: one word on average. In addition, the yeast lexicon listed fewer synonyms on average (1.86). These factors may account, in part, for the better performance of systems on yeast gene identifiers. However, the statistics on synonym length, number of synonyms per identifier, and number of identifiers per synonym were all computed relative to the lexicons supplied by the task organizers. These lexicons were derived automatically from the appropriate model organism database or other resource (for example, Entrez Gene) and varied in quality and completeness. Many groups, particularly the top-scoring groups, compensated for this by enriching the lexicon, or pruning it of ambiguous terms, or both. The statistics for human genes appear roughly comparable to those for fly and mouse in complexity, although the human gene lexicon contained approximately twice as many synonyms as mouse or fly, which can increase coverage (recall) but hurt precision, by increasing ambiguity.

**Table 2:** Statistics comparing BioCreative II gene normalization task (human) with BioCreative I tasks (mouse, fly, and yeast).

	Number of unique IDs	Average synonym length in words	Average synonyms per identifier	Average identifiers per synonym (ambiguity)	BioCreative maximum recall @ precision	BioCreative maximum F-measure
Human	32,975	2.17	5.55	1.12	0.88 @ 0.50	0.81
Mouse	52,494	2.77	2.48	1.02	0.90 @ 0.43	0.79
Yeast	7,928	1.00	1.86	1.01	0.96 @ 0.65	0.92
Fly	27,749	1.47	2.94	1.09	0.84 @ 0.73	0.82

Statistics on synonyms are based on lexical resources provided by the task organizers.

An interesting statistic is the maximum recall reported among the systems. This number gives some indication of whether there is a 'recall ceiling' inhibiting progress - that is, whether there are mentions that are hard to map to their full forms or their identifiers. There are multiple sources of recall problems.

• Conjoined expressions and range expressions where there may be no explicit trace of a gene mention. For example, the expression *freac-1 to freac-7 *implies mention of *freac-2*, but this must be deduced by the processing of the range expression.

• Short, highly ambiguous symbols that can stand for multiple genes, e.g., *AMP*.

• Long names that are descriptions of the gene or paraphrases or permutations of the gene name found in the lexicon - for example, 'receptor for ciliary neurotrophic factor' as compared with the form in the lexicon, namely 'ciliary neurotrophic factor receptor'. Many of the BioCreative II teams used various kinds of fuzzy matching to address this last problem.

The highest recall on an official result for human genes was 0.875. However, an analysis of all of the submitted results reveals that there were only 22 out of 785 identifiers that were missed by all of the systems (2.7%), for a surprisingly high 'pooled maximum recall' of 97.2% (at 23.2% precision). This is important, because it indicates that there is no 'recall ceiling'. Further progress will come from better disambiguation, to improve precision without losing recall.

These 22 'missing' gene identifiers are shown in Additional data file 3, together with a short description or diagnosis of the problem. Of the 22 missing genes, which were distributed across 16 abstracts, four genes were mentioned with limited local context (for example, 'cofactor A'), four were mentioned using complex descriptions, 11 were problematic because of missing synonyms in the lexicon, four were problematic because of name ambiguity (sometimes together with missing synonyms), and one was due to a conjoined expression (insulin- and EGF-receptor).

### Improvement through combining multiple systems

Table 3 shows the tabulation of results for different levels of consensus, using each team's top micro-averaged run. Each identifier/PMID pair was tabulated based on the number of systems that included it in their submission, and further classified based on whether it matched the gold standard (true positive) or not (false positive). Column 1 is the level of consensus (number of 'votes'), and columns 2 and 3 are the number of false positives and true positives returned at that level of consensus. For example, row 1 shows that one false positive and 86 true positives were returned by all 20 runs. Recall, precision and F-measure were calculated based on identifiers returned with at least that many 'votes'. For a 50% consensus level (10 or more 'votes'), the overall recall for the composite system was 79.4%, the precision was 92.4%, and the F-measure was an impressive 85.4%.

**Table 3:** Number of false positives and true positives at different levels of consensus from best micro-averaged runs of the 20 teams

Votes	Count FP	Count TP	Precision	Recall	F-measure
20	1	86	0.989	0.110	0.197
19	3	204	0.986	0.260	0.411
18	7	288	0.976	0.367	0.533
17	8	359	0.978	0.457	0.623
16	11	421	0.975	0.536	0.692
15	13	470	0.973	0.599	0.741
14	15	513	0.972	0.654	0.781
13	19	555	0.967	0.707	0.817
12	30	572	0.950	0.729	0.825
11	42	599	0.934	0.763	0.840
10	51	623	0.924	0.794	0.854
9	77	644	0.893	0.820	0.855
8	103	667	0.866	0.850	0.858
7	130	685	0.840	0.873	0.856
6	160	704	0.815	0.897	0.854
5	221	714	0.764	0.910	0.830
4	304	721	0.703	0.918	0.797
3	435	743	0.631	0.946	0.757
2	713	751	0.513	0.957	0.668
1	2522	763	0.232	0.972	0.375
Total		785			

The table shows cumulative number of false positives and true positives (columns 2 and 3) obtained for a given level of consensus (column 1) from the top micro-averaged run of each team. Recall, precision, and F-measure were calculated using the consensus level as the minimum number of votes needed to include an identifier as an 'answer'. The total under True Positive Count indicates that there were 22 true positives that no system identified; see additional data file 3 for a listing of these.

Analysis of these results gives some insight into what is easy and what is hard. For example, of the 86 genes that all 20 systems identified, 62 were short forms (single words or symbols). The single false positive identified by all runs was a mention of Notch1, which described experimental results in mouse (PMID 9315665).

Given these results from a simple voting procedure, the next step was to determine whether overall performance could be improved by combining results in a more sophisticated way. To evaluate this, we trained two common binary classifiers: naïve Bayesian classifiers and support vector machines (SVMs). The classifiers used a Boolean feature vector for each run, based on whether a particular pair of PubMed/MEDLINE and Entrez Gene identifiers was part of that submission. For this 'system combination' experiment, we selected the best macro-averaged submission from each team and performed 10-fold cross-validation on the test set, training the classifiers separately for precision, recall, and F-measure. The results are shown in Table 4 for both the naïve Bayes classifier and the SVM (standard deviation given in parentheses). The SVM runs achieved an average F-measure of 0.92 (all systems combined) and 0.88 (top five systems combined), both of which exceeded the best run reported for any single system.

**Table 4:** Results of 10-fold cross-validation on classifiers trained on the pooled submissions

	Recall	Precision	F-measure
Best macro averaged submission from each group			
Naïve Bayes	0.92 (0.046)	0.75 (0.036)	0.83 (0.028)
Support vector machines	0.88 (0.049)	0.96 (0.026)	0.92 (0.031)
Best macro averaged from top 5 ranked groups			
Naïve Bayes	0.91 (0.062)	0.75 (0.062)	0.82 (0.060)
Support vector machines	0.82 (0.054)	0.91 (0.041)	0.86 (0.040)

These results suggest that different systems are making different mistakes, and that a weighted combination of results from multiple systems can yield a significant boost in performance. It also suggests that lower-ranked systems are making significant contributions. This observation is corroborated by examining the list of gene identifiers identified correctly by only one out of 20 systems (Additional data file 4). There were 12 such identifiers, contributed by 10 systems; 7 of the 12 contributions came from systems in the bottom half of the rankings.

## Discussion

The different teams approached the GN task from a variety of angles. The *Materials and methods *section (below) contains brief overviews of the specific approaches of the different teams; the workshop proceedings contain more extended descriptions of the individual systems. In this section, we highlight some of the commonalities and some of the successful approaches that were used in the evaluation.

For overview purposes, we can break down the GN task into three basic tasks.

• Preprocessing of the text to regularize it and to identify linguistic units such as words and sentences, and even categories of words and phrases, such as mentions of genes and gene products. This step could also include special handling for prefixes, suffixes, and enumerations or conjunctions.

• Generation of candidate gene identifiers, generally by associating text strings (sequences of words in the text) with identifiers, using a lexicon.

• Pruning of the list of candidate identifiers to remove false positives and to disambiguate in cases where a mention could be mapped to more than one identifier.

Not all teams followed this approach. For example, Team 14 [9] did no linguistic preprocessing; they generated candidate gene identifiers using a text categorization approach, and followed this step by identifying text evidence for the selected gene/protein identifiers. Other teams (7 and 42) avoided the tokenization step and relied on matching against a lexicon to find candidate gene identifiers.

### Preprocessing and linguistic processing

A number of teams built directly on their BioCreative GM system (teams 4, 6, 104, 109, 110, and 111) to handle this process. Several teams (teams 4, 36, 101, and 107) used 'off-the-shelf' systems such as LingPipe [10] or ABNER [11] for recognition of gene mentions, followed by various postprocessing steps, and several teams also combined results from multiple gene mention systems (teams 4 and 107).

Several teams incorporated a special-purpose module for handling abbreviations and gene symbols (teams 4, 13, 34, 36, and 104). In some systems, gene symbols were processed via a separate pipeline; in others, any three-letter expression was checked for an adjacent full form, and then both forms were used in subsequent term matching.

Four teams (teams 4, 34, 42, and 109) discussed handling of conjoined forms or enumerations, such as 'protein kinase C isoforms alpha, epsilon, and zeta' or 'freac-1 to freac-7'. Team 4 noted in their write-up [12] that an estimated 8% of the names in the development data involved some form of conjunction. The explicit handling of conjoined forms represented a significant advance in BioCreative II, as compared with the first BioCreative.

### Candidate generation

This stage associates text strings with specific gene identifiers, based on matching the strings against a lexicon. The lexicon contains, for each gene identifier, a set of associated names for the gene, including the official name, the official gene symbol, and other 'aliases' or synonyms. There are complex trade-offs between enriching the lexicon and using a loose or fuzzy matching procedure to associate gene identifiers with text strings.

The organizers provided the participants with a 'starter' lexicon, as described in the *Materials and methods *section (below). Many teams chose to expand the lexicon with additional resources, or to prune the lexicon by removing highly ambiguous terms, or both. Lexicon expansion was done through the addition of further synonyms (teams 13, 42, 101, 108, and 109) and pattern-based expansion of the lexicon, such as adding variants for Greek as well as Roman suffixes; see particularly teams 4, 7, 13, and 34, although other teams handled this during preprocessing or through a fuzzy matching stage. Some teams (13, 34, and 113) pruned the lexicon by eliminating highly ambiguous terms or terms that generated false positives, such as common English words or biological terms that were not gene names but occurred in similar contexts, such as cell lines. Teams 4, 13, 34, 109, and 113 explored performance results using different lexicon variants. Interestingly, team 113 reported higher results (in particular, higher precision) using a smaller, carefully edited lexicon.

Many teams paid particular attention to the procedure for matching sequences of words in text against terms in the lexicon. Techniques included minimum edit distance (team 108), Dice coefficient (teams 4 and 36), Jaro and Jaro-Winkler distance (teams 6 and 111), and TFIDF and softTFIDF (teams 42 and 111), as well as percentage of matching words and matching against regular expressions. One team (107) used trigram matching: each candidate gene mention was reduced to character trigrams, which were matched against a lexicon.

### Disambiguation and removal of false positives

False positives can arise in several ways. Two identifiers can have synonyms that match or partially match a mention in text, as was discussed earlier for the terms 'Humly9' and 'Ly9'. The mention can also be a word or phrase in English (or in biology), in addition to being a gene name or symbol, for example 'per' or 'period'. Another source of ambiguity is the use of a single gene name used across multiple organisms (as in the case of the false positive for 'Notch1' discussed above). Compared with participants in BioCreative I, the systems from BioCreative II focused much more on this stage. For several teams (teams 6, 58, 101, 109, and 111), the disambiguation and filtering step was implemented as a classification step, where a classifier was trained to distinguish valid gene identifiers from spurious ones, as done in BioCreative I [13]. Other groups used additional resources and vector-based context models to select among ambiguous terms (teams 14, 34, 42, 107, and 108). Still other teams relied on heuristics to remove potential false positives, including stop word lists for ambiguous or non-gene terms, and filters for nonhuman gene names (teams 4, 7, 13, 30, and 34).

## Conclusion

Performance on the BioCreative II GN task demonstrates progress since the first BioCreative workshop in 2004. The results obtained for human gene/protein identification are comparable to results obtained earlier for mouse and fly; three teams achieved an F-measure of 0.80 or above for one of their runs. However, there is significant progress along several new dimensions. First, the assessment involved 20 groups, as compared with eight groups for BioCreative I. The results achieved by combining input from all of the participating systems outperformed any single system, achieving F-measures from 0.85 to 0.92, depending on the method of combination.

The participating teams explored the 'solution space' for this challenge evaluation well. Four teams incorporated explicit handling of conjunction and enumeration; this no longer seems to be a significant cause of loss in recall. The 'maximum recall' system achieved a recall of 96.2% (precision 23.1%). A number of groups did contrastive studies on the utility of adding lexical resources and contextual resources, and on the benefits of lexicon curation. The participants also explored novel approaches to the matching of mentions and lexical resources, and there was significant exploration of contextual models for disambiguation and removal of false positives. An interesting finding was that many groups did not feel the need for large training corpora, especially those using lexicon-based approaches.

What does this mean in terms of practical performance? Performance depends on a number of factors: the quality and completeness of the lexical resources; the selection criteria of the articles, including date, journal, domain, and whether they are likely to contain curatable information; the amount of both intra-species and inter-species gene symbol ambiguity; the types of textual input (abstract, full text) and the types of preprocessing required in particular for full text articles; and quantity of data to be handled (all of PubMed/MEDLINE versus specialized subsets).

The formulation of the BioCreative II GN task is still quite artificial. A more realistic task would be to extract and normalize protein names across multiple species, from full text articles, such as was required for the PPI task.

Despite these limitations, normalization technology is making rapid progress. It has the potential to provide improved annotation consistency for gene mention linkages to databases, more efficient updating of existing annotations, and, when applied across large collections, more focused gene-centric literature search.

It is always important to evaluate the evaluation. Criteria for a successful evaluation include participation, progress, diversity of approaches, exchange of scientific information, and emergence of standards. We can see all of these happening in the BioCreative evaluation. There is enthusiastic participation across the entire range of BioCreative tasks. The research community is making significant progress, as shown by the larger number of high-performing systems. There are more groups engaged, and more teams are emerging that combine skills from multiple disciplines, including biology, bioinformatics, linguistics, machine learning, natural language understanding, and information retrieval. There is a healthy variety of approaches represented. We are seeing exploration of ideas developed in the first BioCreative, such as use of a high-recall gene mention 'nomination' process, following by a filtering stage. Also, although the GN task was designed to leverage existing standards, such as Entrez Gene identifiers, we are seeing the emergence of reusable component-ware, and a number of high-performing systems that are taking advantage of this. As we go forward, the BioCreative Workshop will provide an opportunity to exchange insights and to define the next set of challenges for this community to tackle.

## Materials and methods

### Data preparation

We handled the data preparation for this task following many of the same procedures developed for the first BioCreative GN task [2]. There were, however, some differences described in greater detail in [14]. We used the GOA annotated records as the basis for selecting documents rich in human genes and proteins. However, the GOA annotators annotate from full text, and we were using only abstracts; furthermore, the GOA annotation process does not include every human gene mentioned in an article, but only specific genes of interest. Finally, because we wished to provide a richer linguistic context for the dataset, the BioCreative annotators were asked to flag one string in the text that represented the mention of each annotated gene. This had the effect of supplying a short 'evidence passage' for each gene identifier in the abstract. For the evaluation, we required that submissions provide a text string that gave rise to the selected Entrez Gene identifier in the abstract.

As described in [15], we used the file gene_association.goa_human (from the Gene Ontology homepage [16] downloaded on 10 October 2005) to provide 11,073 PubMed/MEDLINE identifiers (and 10,730 abstracts) associated with journal articles likely to have mentions of human genes and proteins. We then used the file gene2pubmed obtained from the National Center for Biotechnology Information (NCBI) [17] on 21 October 2005, along with the Gene Ontology (GO) annotations, to create a large set of partially annotated abstracts. We set aside 5,000 abstracts as a noisy training set, which we made available to the participants, along with a lexicon of gene identifiers and the corresponding names and gene symbols. From the 5,730 remaining abstracts, we randomly selected 600 abstracts for expert annotation. The resulting annotations consisted of the list of unique Entrez Gene identifiers for the human genes and gene products in each abstract, plus the text snippets.

To create the training and test sets, an expert annotator performed a detailed manual annotation of the abstracts. The annotator also flagged any annotations about which he had a question. These were checked by the first author. To verify the quality of the annotation, we performed a small inter-annotator agreement study. It is important to understand the quality of the annotated data, because this may limit performance of the systems that train on the data. In addition, it provides a ceiling for system performance. For the study, two annotators independently annotated the first 30 abstracts in the training set. There were 71 identifiers in common and 7 differences, for an overall interannotator agreement of 91% [14]. The final training set consisted of 281 abstracts; the blind test set consisted of 262 abstracts.

We did further *post hoc *validation of the gold standard by looking at gene identifiers for which a majority of system outputs differed from the gold standard. The submissions were scored using the preliminary answer key (original gold standard); we then selected the results of the top-ranking (micro-averaged) submission from each team. We pooled the results and re-examined any annotation where over 50% of the groups disagreed with the gold standard. This led us to reexamine 219 annotations in 126 abstracts. As a result, we added 32 annotations and removed 21 annotations. The final gold standard contained 785 gene identifiers in 262 abstracts, with an interannotator agreement of 96.7% compared to the original gold standard.

### Lexical resources

In addition to the annotated abstracts and the noisy training data, we also provided participants with a lexicon. To create the lexicon, we took the gene symbol and gene name information for each human Entrez Gene identifier from the gene_info file from NCBI [17]. We merged this with name, gene and synonym entries taken from UniProt [18]. Suffixes containing '_HUMAN', '1_HUMAN', 'H_HUMAN', 'protein', 'precursor', or 'antigen' were stripped from the terms and added to the lexicon as separate terms, in addition to the original term. The Hugo Gene Name Consortium (HGNC) symbol, name, and alias entries were also added [19]. We then identified the most often repeated phrases across identifiers as well as those that had numerous matches in the 5,000 abstracts of noisy training data. We used these to create a short (381 term) 'stop' list to remove the most common terms that were unlikely to be gene or protein names but which had entered the lexicon as full synonyms, for example 'recessive', 'neural', 'Zeta', 'liver', and so on; see the BioCreative CVS archive [20] for the full list. The result was a lexicon of 32,975 distinct Entrez Gene identifiers linked to a total of 163,478 unique terms. The majority of identifiers had more than one term attached (average 5.5), although 8,385 had only one.

### Scoring and revising the gold standard

Scoring was done with a Python script that matched the gene identifiers returned for each abstract against the gold standard for that abstract. The script also checked for the presence of a textual evidence string for each gene, although this did not affect the actual score, and the textual evidence provided by many submissions did not exactly match the original abstracts. The scoring software was provided to the participants, along with the answer key for the training data.

### System descriptions

This section contains brief system descriptions of the participating teams, presented in rank order of the micro-average (see Table 1).

#### Team 42 (Jörg Hakenberg, Technische Universität Dresden)

The system [21] used the provided lexical resource plus additional synonyms found in Entrez Gene's 'Other designations' field. Synonyms were sorted into four categories: database identifiers ('Kiaa0958'), abbreviations ('ICAM-1'), compound names ('RNA-helicase alpha'), and spurious names ('AA'). Spurious names were removed from the lexicon. Each remaining synonym was preprocessed to generate a regular expression that matched as many variations as possible. Database identifiers (KIAA, HGNC, UniProt, and so on) appeared in few variations, and fixed rules were used to transform different types of database IDs into regular expressions. Abbreviations were split at every transition between upper or lower case letters, symbols, or digits; for example, 'CD95R' became 'CD 95 R'. Following this, variations for every single element were generated, for example 'CD, Cd, cd' and 'r, Receptor, receptor', depending on the element and its position. This also allowed for changes in Greek/English lettering and Arabic/Roman numbering. Tokens of compound names were handled like abbreviations. The resulting set of regular expressions was matched against the text, with the requirement that a match be surrounded by word boundaries (interpunctuation, bracketing, quotation marks, hyphens, or slashes). The system did not tokenize texts or stem words. Every text was preprocessed to find abbreviated enumerations of multiple (potential) gene names ('LERK-2 and -5'.) Such occurrences were replaced with their respective fully expanded forms ('LERK-2 and LERK-5').

After the initial recognition, which assigned candidate IDs to each potential name, highly ambiguous and unspecific names were filtered using TF*IDF scores and another regular expression to remove general terms referring to tissues, functions, molecules or a combination of those ('liver', 'calcium', 'anion', 'channel'). TF scores were based on the current abstract, and IDF was based on the noisy training data; both single and multiword names were removed if they fell below a threshold (indicating highly ambiguous terms). Filtering also took place to remove references to genes in other organisms, based on an analysis of the immediate context of a gene name for species and cell lines; this removed mentions like 'rat ATP synthase OSCP', but left phrases like 'human and mouse fibulin-2'.

The final disambiguation of names used gene context models extracted from the Entrez Gene entry for each gene, as well as UniProt and GOA entries for proteins encoded by the gene. In addition to each gene's Entrez Gene summary, a gene context model stored information on GO annotations, functions, tissue specificity, locations, mutations, GeneRIFs, domains, interaction partners, UniProt keywords, and diseases obtained from all three resources. Textual sources were scored against the current abstract using either cosine similarity or a normalized overlap count. For GO terms, GoPubMed [22] was used to associate each abstract with GO terms. This set was then compared with the set of GO terms for each candidate gene. The similarity measure was based on the absolute distance of two GO terms (path length via the lowest common ancestor [LCA]) and the depth of the LCA; low-level terms would thus score better than high-level terms. The combination of all scores, with weights adjusting importance/reliability of data types, defined an overall confidence. All candidate IDs were ranked according to this confidence and the top ID was selected if it scored above a certain threshold.

#### Team 34 (Katrin Fundel, Ludwig-Maximilians-Universität München)

Team 34 made use of a carefully curated synonym dictionary and previously existing matching approaches [23], which were expanded by rule-based post-filtering and a general approach for disambiguation [24].

A large synonym dictionary for human gene names was compiled from Entrez Gene, Swiss-Prot, and HGNC. The dictionary was automatically curated to increase precision and to obtain high recall. Gene and protein names were identified in the texts by application of exact matching and approximate matching as implemented in ProMiner [25,26] and merged into one set of matches for the subsequent steps. Subsequently, matches were post-filtered based on their context. A rule-based post-filter was used for resolving enumerations, pruning matches of gene names occurring close to organism names other than human, and pruning matches of gene names where the text referred to cell lines, cell types, gene families, pathways, chromosomal locations, amino acids, and so on.

For term disambiguation with respect to non-gene terms and within the synonym dictionary, a general approach was applied based on the similarity between text fragments and dictionary entries. A rule-based approach was applied to extract short form/long form pairs of abbreviations from the texts; these abbreviations were combined with a public abbreviation dictionary and all non-gene, non-protein concepts of the Unified Medical Language System (UMLS). Based on this combined dictionary, a dictionary of 'alternative concepts' (non-gene or non-protein concepts) was compiled by retaining only entries that did not exceed a certain similarity with any of the entries of the gene name dictionary. For this purpose, the individual dictionary entries were represented as feature vectors, in which the individual features represented word-stems or character trigrams, weighted by their inverse document frequency. Terms can have both gene and non-gene meanings or can be ambiguous between two or more genes. The same approach was used for resolving both types of ambiguity; the similarity between all noun phrase chunks from a given abstract and alternative synonyms of the possible genes/concepts was determined and the object yielding the maximal cosine similarity was reported, provided this similarity was achieved by only one gene/concept and was above a certain threshold.

Given that many gene names are ambiguous and overlap with non-gene terms and abbreviations, disambiguation plays an important role in gene normalization. The results demonstrate that the proposed disambiguation approach achieves good performance. As the approach directly exploits the information contained in the dictionaries, it does not require annotated training data. Given the large number of ambiguous terms and the constantly evolving nomenclature, this is an important advantage.

#### Team 13 (Juliane Fluck, Fraunhofer Institute for Algorithms and Scientific Computing [SCAI])

The ProMiner system [25,26] relies on an approximate string matching algorithm in conjunction with an automatically curated dictionary. In an initial preprocessing step, additional spelling variants were added automatically to the dictionary and external dictionaries were used for the removal of non-gene names. To improve the precision of the search, ambiguous names were detected using the dictionary, an abbreviation dictionary, or the frequency of occurrence of words in the text corpus.

The dictionary for human genes/proteins was extracted from the description fields of human Entrez Gene entries as well as human UniProt entries. All entries that mapped transitively to each other in the International Protein Index were merged into a single dictionary entry.

Automatic dictionary curation drew on several information sources, including acronym expansion and addition of spelling variants or filtering synonyms on the basis of regular expressions. Additionally, a biomedical terminology dictionary was used for curation of the human dictionary; this was extracted from OBO ontologies [27] for disease, tissue, organism, and protein family names and from a manually curated list generated through inspection of various training corpora in different former and ongoing projects. Finally, an acronym dictionary with gene-specific short forms and non-gene long forms was used as an additional dictionary for disambiguation purposes.

In a compilation step, information such as frequency in a reference corpus, inclusion in a common word dictionary, or the acronym dictionary was used to classify synonyms into one of several classes.

The search system was based on an approximate string matching algorithm supporting not only exact matches but also small variations in spelling. The different synonym classes were searched with specific parameter settings such as case sensitive, exact, or permuted search. To recognize names split by an insertion of acronyms, for example 'coenzyme A (HMG-CoA) synthase', additional runs after removal of the brackets or the full bracketed expression was done and the results were merged. This led to improved performance on the training set but not on the test set.

Synonyms contained in more than one Entrez Gene entry or additionally found in the acronym dictionary were labeled as ambiguous. Matches on ambiguous synonyms were only accepted if an unambiguous synonym for the same entry was found. The best results of the system were achieved with this setting. Accepting ambiguous hits led to an increase in recall but an overall decrease in F score. Using a co-occurrence filter for organism names decreased the recall and the overall performance.

#### Team 4 (Zhiyong Lu, University of Colorado School of Medicine)

Six gene mention (GM) tagging systems were combined using a filter, designed for the GM task, that favors recall, using the longest mention in case of overlaps. The systems were KeX [28], AbGene [29], the team's BioCreative I GM system [30], LingPipe [10], and ABNER (NLPBA and BioCreative models) [11]. Heuristics removed false positives by identifying amino acids, protein families or domains, and nonhuman proteins. Tokenization included extracting individual gene names from conjoined structures, for example from *IL3*/*IL5 *to IL3 and IL5, and from *freac1-freac7 *to freac1, freac2 ... freac7.

For the lexicon, gene symbols, synonyms, and full names were extracted from the Entrez Gene database for all human genes. The contents were then regularized with a set of heuristics, including Roman letters to Arabic numerals, Greek letters to single letters, words to lowercase, removal of parenthesized text, removal of punctuation, removal of spaces, and removal of terms only one character long.

Gene mentions found in the text were regularized using the same set of heuristics used for dictionary construction. Exact string matching was then used to find matches between the gene mentions and the dictionary. If a single match was found, then the identifier was returned. Disambiguation was performed if multiple matches were found. The first approach used the Schwartz and Hearst algorithm [31] for detecting abbreviation definitions in biomedical text. The second approach used the five preceding and five trailing tokens surrounding the gene mention. In both cases the Dice coefficient between the extracted text (abbreviation or flanking tokens) and the full name of each gene candidate as given in the lexicon was computed, and the gene with the highest nonzero Dice coefficient was selected. Further removal of false positives was achieved through the use of custom stop lists: a list containing 5,000 words derived by word frequency in the Brown corpus; a list containing protein family terms; a list containing non-protein-indicating terms; and a list of small molecules.

#### Team 109 (Hongfang Liu, Georgetown University Medical Center)

The base gene/protein name normalization system included three modules. The first module was lexicon-lookup, where the lexicon consisted of terms associated with human Entrez Gene records. The second module used machine learning to integrate the results of the gene/protein name mention tagger [32], name sources, name ambiguity, false positive rates, popularity, and token shape information. The third module used a similarity-based method to associate Entrez Gene records with long phrases detected by the gene/protein name mention tagger.

The lexicon was compiled from terms for human genes from Entrez Gene, Online Mendelian Inheritance in Man, HGNC, and BioThesaurus [33]. The synonymy relationship was based on rich cross-reference information provided by Entrez Gene and UniProtKB. All terms were then normalized by changing to lower case, ignoring punctuation marks, and transferring words to their base forms according to the UMLS Specialist Lexicon. The same normalization procedure was applied to each document, followed by longest string matching lookup. If the string contained specialized patterns, which usually were abbreviated forms for several entities from the same family (for example, 'HAP2, 3, 4' or 'HAP2-4', 'HAP-2, -3, and -4', or 'HAP2/4'), then they were separated and reassembled into distinct strings with their own mappings. For example, 'HAP2/4' would become two strings, 'HAP2' and 'HAP4'. This stage returned a list of pairs (Phrase, EGID), in which Phrase was a text string mapped to a lexicon entry and EGID was the Entrez Gene identifier. Each pair (Phrase, EGID) was then transformed into a feature vector, and machine learning was used to classify the pair as valid or invalid. The features included the following:

• Phrase-specific features: the gene/protein mention tagger result, the ambiguity of Phrase, the number of occurrences of Phrase in the document, the number of occurrences of Phrase in the top one million words provided by MedPost, and some typographic features.

• EGID-specific features: the number of different strings mapped to EGID and their occurrences in the text.

• (Phrase, EGID)-specific features: a metric to measure the association power between Phrase and EGID based on the lexicon, a Boolean feature indicating whether Phrase could be mapped to EGID through exact string matching, and the false positive rate of the pair in the training set.

Names with multiple words in a lexicon may appear in the text with some of the words missing, or in different word orders or forms. The system incorporated a similarity-based method for normalizing names detected by the gene/protein mention tagger. The number of overlapping words was counted between phrases detected as entity names in text and names in the dictionary. If over 90% of the words in a name from the dictionary were found in the names detected by the gene/protein name tagger, the names in the text were normalized to associated record(s) of the name.

#### Team 104 (Rafael Torres, Bioalma, Tres Cantos, Madrid, Spain)

TextDetective [34] distinguishes between functional gene descriptions (names that describe the function of the gene/protein, for example 'thyrotropin releasing hormone receptor') and symbols (generally abbreviations, for example 'TRHR'). In the case of descriptions, the morphology and semantics of the words are highly indicative. For symbols, the system uses contextual information (the adjoining words that are related to genes and proteins) to detect gene names.

First, sentence boundaries are detected and tokens are assigned to specific classes, such as 'Keyword', 'Stop_word', 'Biological_locations', or 'Type', to select candidates for gene names. For gene symbols, both the local context (the words around a potential symbol) and global context (taking into account all of the occurrences of a symbol in PubMed/MEDLINE) are evaluated. The local context uses a general model that distinguishes genes from non-genes. In the global context, a specific model is generated for each potential symbol. The model reflects how frequently a symbol is used to refer to genes or to other types of entities. This allows us to estimate the 'risk' of tagging a symbol as a gene. The system then attempts to assign the candidate names to an entry in the database. The TextDetective dictionary is used; it contains the entries for human genes from both Entrez Gene and Uniprot.

For gene descriptions, a set of rules is applied to select the dictionary definitions of genes that match the descriptions found in the text. No further disambiguation is performed. If more than one database entry is selectable, no results are returned.

For symbols, all of the entries in the dictionary that contain this symbol are selected. Where there is more than one possible candidate, a disambiguation process is performed to select the most suitable entry. This process uses a list of 'keywords' for each gene with a weighting to represent its importance. These words are extracted from the functional annotations, GeneRIFs, and summary sections in Entrez Gene and UniProt. For each candidate, the system obtains the sum of the keyword weightings that appear near the name in the text, and selects the one with the highest score. However, the detection is only considered to be correct if the value exceeds the minimum risk value associated with the risk factor for the symbol.

The most relevant parameters controlling the trade-off between precision and recall are as follows:

• The importance given to the risk factor: higher values increase precision because more ambiguous symbols are rejected; if the value is decreased, then recall takes priority.

• The number of words that are analyzed in the context of a gene symbol: larger 'windows' increase recall because words further away from the name are taken into account and 'good' words are more likely to be found; smaller windows favor precision.

• The keywords and their associated weightings: the higher the threshold, the more keywords have to be present close to the gene name to assign it to a specific database entity; high values favor precision but decrease recall.

#### Team 101 (Heng-hui Liu, National Cheng Kung University)

The system [35] consisted of two major components. The first component recognized candidate gene mentions in text, and then matched these mentions against a lexicon to assign corresponding identifiers. If a mention was associated with more than one identifier, then the ambiguous mention was forwarded to the second component for removal of identifiers of lower confidence.

The first component recognized candidate mentions in text. Three available Named Entity Recognition packages were evaluated on the BioCreative Named Entity training data, namely LingPipe, ABNER, and BioNLP, and LingPipe was selected.

Entrez Gene and BioThesaurus were used to establish the lexicon, extracting the entries for human genes/proteins from these two resources and removing redundant entries.

To handle morphological variation, the system used several normalization rules, including normalization of case, replacement of hyphens with space, removal of punctuation, and removal of parenthesized material.

The disambiguation component filtered out identifiers with a lower degree of confidence. The degree of confidence was determined by the relations between genes and key words in text (such as MeSH terms or GO terms) and specific identifiers. A maximum entropy model was used to learn the relationships from training data. Because of the insufficiency of the BioCreative II training dataset to cover all human genes, NCBI's 'gene2pubmed' was used to collect about 60,000 articles for training. After training, given a text and an ambiguous gene mention, a model assigned probabilities to candidate identifiers; these values could be used to rank these identifiers. Two kinds of feature functions were considered for training, each one with its own advantages. Therefore, these models were combined. Using ideas of fuzzy set aggregation, models act as membership functions that convert observation (occurrence of key words) into membership degrees (probabilities) of the context supporting a certain identifier. For example, given a text *c *and a mention *g*, which associates with identifiers *id*_1 _and *id*_2_, through model *f*_*m*_, g can be represented as:

*f*_*m*_(*c*, *g*) = {*id*_1_(0.6), *id*_2_(0.4)}

where 0.6 and 0.4 are degrees of *c *supporting *id*_1 _and *id*_2_, respectively. Finally, an ordered weighted averaging operator was used to combine results from different models.

#### Team 107 (Michael Krauthammer, Yale University School of Medicine)

The approach [36] was based on the idea of reusing existing programs for gene name recognition and classification (entity recognition), with primary focus on the task of mapping those names to Entrez Gene IDs. For entity recognition, the system used ABNER [11] and LingPipe [37], two programs with excellent recall and precision. The system handled lexical variation by transforming gene names into their unique trigrams, and performing trigram matching against a gene dictionary. For ambiguous gene names, an additional contextual analysis was performed on the abstract containing the name. The approach was formalized as a sequence of matrix manipulations, allowing for a fast and coherent implementation of the algorithm.

A combination of two methods was used to map recognized entities to their appropriate gene identifiers: the Trigram Method, and the Network Method. Both methods required preprocessing, using resources from Entrez Gene, to construct a set of method-specific matrices.

The Trigram Method used an approximate representation of a gene name, by transforming a string into the set of its unique trigrams. The similarity between two gene names is the number of their common trigrams (i.e. the intersection of their sets of trigrams). This approach allowed for the fast mapping of gene names to large gene dictionaries, associating names to their gene identifiers.

Often, gene names are ambiguous, and an additional method was needed to pinpoint the correct gene identifiers. To accomplish this, the Network Method examined the words (context) of the abstract in which an entity had been recognized. The idea is as follows. Assume that the Trigram Method determines that a recognized entity *E*, appearing in abstract *a*, can be mapped to two different gene identifiers (genes *X *and *Y*) with equal similarity scores. The network method compares the abstract *a *with a collection of abstracts in which gene *X *and *Y *have already been positively identified. If the content of abstract *a *is closer to the set of abstracts linked to gene *X*, then entity *E *is labeled with gene identifier *X*.

There were several noteworthy features of this approach. First, it separated the local (trigram-based) and contextual mapping, enabling the experimental examination of both processes individually. Second, the local analysis was fast and efficient, avoiding the traditional string matching techniques, which were replaced by simple matrix manipulations. The system achieved an F-measure of 0.76 for the GN task.

#### Team 113 (Martijn Schuemie, Erasmus MC University Medical Center)

The Peregrine system [38] was tested in two separate runs: with the lexicon provided by the BioCreative II organizers, and with a lexicon constructed by combining five different gene databases [39]. Four editing techniques were applied to both: manually checking the 250 most frequently occurring terms in a random subset of PubMed/MEDLINE for ambiguous and erroneous terms; automatic generation of spelling variations; automatic removal of terms consisting only of nonspecific tokens, such as stop-words or numbers; and automatic removal of family names.

Sequences of letters and/or numbers were considered tokens. A term in the text was matched to the lexicon when all tokens of a term were found in order.

Disambiguation was done by a set of simple rules. First of all, a term was considered ambiguous if it was short, did not contain a number, or referred to more than one gene in the lexicon. Ambiguous terms were only matched by the system if the term was the preferred name of a gene, or if a synonym or a highly specific part of a synonym was also detected in the text.

The Peregrine system was designed with two goals in mind. First of all, it should be easy to maintain. There is only a single step (manual check of highly frequent terms) that requires human involvement when implementing a new lexicon. The second goal was speed. Because Peregrine does not rely on part-of-speech tagging or natural language parsing, it is very fast: 100,000 PubMed/MEDLINE records can be processed in 213 seconds on a single machine. The whole of PubMed/MEDLINE can be processed within a single day.

#### Team 108 (William Lau, Center for Information Technology, National Institutes of Health)

The lexicon was created by combining data from the HUGO and Entrez Gene databases without any additional pruning. The algorithm [40] divided the GN task into two major steps. The goal of the first step was high recall. Using a set of regular expression rules, gene symbols were detected using pattern matching. For gene names, an approximate term matching technique was employed. A name was broken into individual tokens, each matched independently. Subsequently, the phrase containing the most tokens was identified. If the ratio between the number of tokens in the candidate and the total number of tokens to be matched exceeded a threshold, the candidate was passed to the second step.

In the second step, a set of statistical and heuristic features was used to measure the level of confidence for each mention extracted. The goal of this step was to reduce the number of false positives. 'Uniqueness' was an estimate of the probability that the candidate was referring to something other than the gene in question. If the mention had a very high frequency of occurrence in the literature, then the score was reduced accordingly, as frequently occurring terms were more likely to have multiple meanings. Another important feature was 'inverse distance', which used edit distance to calculate the similarity between the candidate mention and the corresponding gene term in the database. The 'coverage' feature preferred long mentions over shorter ones, in terms of both the number of matched tokens and the character length of the mention.

There were also several discrete features used to assist the algorithm in selecting the correct identifier in case of ambiguity. First, if more than one unique mention of a gene was extracted from the text, then the confidence that the correct identifier was selected increased. This feature was referred to as 'number of mentions'. In addition, many genes in the Entrez Gene database have not been approved by the HUGO Gene Nomenclature Committee, indicating that the references for these genes may be unstable and that few articles on these genes have been written. Therefore, in the 'official status' feature, preference was given to genes that had been approved. A related feature was 'mention type,' which took into consideration whether the mention was an officially approved term. A 'boosting factor' was also incorporated, to reward or punish a candidate when there was a contextual clue in the citation suggesting whether the mention actually referred to a gene.

The final confidence score was a weighted linear combination of the feature scores, except that the boosting factor was added to the equation as an exponent. In the evaluations, the Nelder-Mead simplex method was used to optimize the set of feature weights on the training data. Consequently, a confidence score was calculated for each gene. If a gene had more than one unique mention in the text, then the maximum score was used. An acceptance threshold could be set to adjust the trade-off between recall and precision.

#### Team 7 (Aaron M Cohen, Oregon Health & Science University)

The OHSU human GN system [41] used an approach similar to the mouse and yeast species system previously reported and evaluated on the BioCreAtIvE 1 dataset [3]. The system used a lexicon automatically extracted from the Entrez Gene database, along with automatically generated orthographic variant expansion using manually generated rules. Because many human gene names, synonyms, and symbols appear orthographically similar to those of mouse genes, it was initially supposed that using the same variant expansion would perform similarly. Unfortunately, human gene name entries are represented differently from those of mouse genes within Entrez. For human gene names, but not mouse, much more information is included within the name and symbol fields. Names often consisted of pairs of comma-separated clauses along with parenthetical expressions. These needed special handling in order to extract strings that would be useful in identifying gene symbols within the biomedical literature. Orthographic variant generation therefore included rules similar to that for mouse, such as replacing spaces with dashes and *vice versa*, as well as human-specific rules, such as removing parenthetical expressions, inverting the order of clauses around commas, and pre-pending an 'h' to short symbols.

Input text was matched against the lexicon using an exact match approach without prior tokenization. The system performed post-match delimiter detection instead of tokenization. This technique avoids some of the complexities of prior tokenization, such as the difficulty of allowing gene symbols to contain delimiters when tokenization is done before matching. The default delimiters allowed included the white space characters as well as single quote, double quote, slash, backslash, parentheses, square brackets, curly brackets, and the following characters:., = ?*!. Overlapping matches were resolved to the longest match.

The default delimiters were all single characters. After examining the training data, it was determined that it would be worth experimenting with multi-character delimiters. Two types were created - inclusions and exclusions. Inclusions are essentially sequences of characters that could delimit a gene symbol. The training data yielded two potential candidates: '-mediated' and '-induced'. Exclusions were text patterns occurring near the matched text that indicated that the match was a false positive. By examining system errors made on the training data, 43 exclusion patterns were identified that improved performance on the training data.

The final stage of the system performed ambiguity detection and removal. Strings that mapped to more than one human gene were resolved to the gene that had the most unambiguous references within the input text (for BioCreative II, the abstract + title). If none of the possible genes had a co-occurring unambiguous reference, no normalized gene reference was generated for that string. This improved precision at some cost to recall. In the officially submitted runs, exclusions improved precision a bit with no cost to recall, while inclusions had little effect. Although better-than-average results demonstrated the value of the overall approach, the difficulty of manually creating effective variant generation and exclusion rules for human genes suggests the possibility of extracting both of these automatically, given a sufficient amount of training data.

#### Team 111 (Chun-Nan Hsu, Academia Sinica)

The system [42] used a high-performing gene mention tagger [43] based on conditional random field models to identify possible gene names. Then TF/IDF and softTFIDF were applied to compute the similarity between a tagged gene name and a synonym in the dictionary. To improve the results of dictionary lookup, an ensemble of classifiers was trained to filter false positives, using string matching scores as the features. The experiments showed that this post-filtering method substantially boosted precision without modifying the dictionary or using any additional external resources.

Before application of TF/IDF, a preprocessing step transformed the string into a token vector and performed case normalization, replacement of hyphens with blanks, and removal of punctuation symbols and parenthesized strings. For softTFIDF, there was no need to perform the preprocessing step because Jaro and Jaro-Winkler with TF/IDF tolerated slight differences between terms in gene names. A list of top ten match scores was returned with the identifier of the top match. A threshold was assigned to filter the outputs of the dictionary lookup.

Based on the match scores, an ensemble of classifiers was applied to determine whether the top ID actually corresponded to the entity. If positive, the ID with its score would be returned; otherwise, the result would be discarded. The feature vector for the ensemble classifier was derived from the top ten match scores of synonyms of ten distinct genes. AdaBoost [44] was used to train an ensemble classifier with this feature set, stopping at 30 iterations. If a dictionary lookup result had a small false negative rate but a large true positive and false positive rate (low precision and high recall), then the classification method would boost the precision as well as the F score. When more than one entry in the dictionary shared a top match score, the system used a tie-breaking strategy, returning the identifier of the entry that maximized the occurrences where the entity appeared as a substring in the synonyms of that entry.

#### Team 30 (Anna Divoli, University of California, Berkeley)

A version of an in-house gene recognition and normalization tool, originally developed for the TREC 2003 Genomics Track, was used for the gene normalization task [45], restricted to the master list of human gene/protein IDs provided by the organizers.

The biggest problem was ambiguity. For example, 'SYT' can refer to two human genes with different identifiers, namely SYT1 (ID 6857) and SS18 (ID 6760). This was addressed using two principles for word sense disambiguation: one sense per collocation (assign a single ID for each gene/protein instance), and one sense per discourse (assign the same ID to all instances of a given gene/protein within a document) [46]. To these was added a third (weak) principle: no synonyms, which assumed that in case multiple names were possible in the literature for a given gene/protein name, in a particular document authors tended to stick to just one of them. This meant that two different gene names/aliases were unlikely to refer to the same gene/protein ID in the same text. One notable exception was when the gene/protein was mentioned for the first time, in which case authors were likely to introduce the correspondence between the full name and the abbreviation, for example 'The dopamine D4 receptor gene (DRD4) shows considerable homology to DRD2.' This issue was not addressed for BioCreative II.

The algorithm was as follows.

• Step 1: Assign the IDs for all unambiguous gene/protein instances (the ones for which there is a single possible ID).

• Step 2: (a) Exclude all IDs recognized so far from all lists of possible candidates. (b) Assign the corresponding ID for all unambiguous gene/protein instances. (c) If there was at least one new assignment, then go to 2(a).

• Step 3: (a) Exclude all IDs recognized so far from all lists of possible candidates. (b) Assign the current instance an ID from the set of the currently available IDs. (c) If there was at least one new assignment, then go to 3(a).

Step 2 considered the instances sorted by length in descending order (long forms first), whereas step 3 sorted them by (1/*I *+ 0.001 × *L*), where *I *is the number of different possible IDs for that instance, and *L *is the instance length (sorted by less ambiguous instances, and then by length).

#### Team 6 (Xinglong Wang, University of Edinburgh)

Team 6 adapted a GN system used in its NLP pipeline [47] for extracting protein-protein interactions from biomedical texts. The system was developed for normalizing proteins but it can also normalize other biological entities (drug compounds, disease types, and experimental methods) without requiring extensive knowledge of the new domain.

The system first uses a gene mention named entity component to mark up entities of types *gene *and *gene product*. A string-distance-based fuzzy matcher then searches the gene lexicon and calculates scores of string similarity between the mentions and lexicon entries using a formula similar to JaroWinkler. The matcher takes into account the commonality and differences in string suffixes, such as Arabic and Roman numbers. Sets of equivalent suffixes are defined (e.g., Roman I = Arabic 1). Strings with common suffixes are rewarded whilst those with different ones are penalized. The value is finally normalized by the length of the string. At the end of the fuzzy-matching stage, each mention recognized by named entity recognition is associated with the single highest-scoring match from the gene lexicon, in terms of the string similarity measure, where each match is associated with one or more identifiers (in cases where ambiguity occurs).

To resolve ambiguity, a machine learning algorithm learns a model to predict the most probable identifier out of a pool of candidates returned by the fuzzy matcher. The machine learning algorithm uses contextual properties surrounding gene mentions such as adjacent words, their part-of-speech tags, and so on, as well as complex features such as NER confidence and string similarity scores between all the mentions in the document and the description associated with the gene identifier. An SVM model was then trained to predict the most probable identifiers for gene mentions.

#### Team 36 (Bob Leaman, Arizona State University)

The gene normalization system implemented for BioCreative II [48] was a lightweight implementation that mixed well-known systems with the initial implementation of new, relatively nonstandard, ideas. Overall, the system relied heavily on orthographic and syntactic information rather than semantic knowledge, including biological domain knowledge. The system had four distinct execution phases, namely extraction, filtering, normalization, and disambiguation, with most of the complexity residing in the normalization phase.

The system was intended primarily to test gene normalization ideas and therefore employed ABNER for tagging gene mentions in each abstract, trained on the BioCreAtIvE 1a data. After gene mentions were tagged and extracted, acronyms were resolved using the Stanford Biomedical Abbreviation database and the provided Java code. The list of gene mentions found was the only data passed from the underlying abstract to the next phase.

In the filtering phase, mentions of generic words (such as 'gene' and 'protein') were dropped. Specifically, the system removed gene mentions consisting entirely of generic words such as organism names, protein types and descriptors such as 'enzyme', 'amyloid', 'protein' and other terms such as 'DNA' or 'alpha.'

The system compared the mention with each of the standard gene names and computed a similarity score for each comparison. This score was based on the Dice coefficient, which weights matches twice and normalizes over the total number of tokens. The baseline system added some weights based on the frequency of token occurrence and origin, but these modifications proved to be detrimental to performance. Tokens were initially considered a match if they contained exactly the same series of characters or represented synonymous ordinal values, such as Arabic and Roman numerals and the letters of the Greek alphabet. Matches with a low score were dropped from further consideration.

Because the normalization phase returned a set of candidate gene names from the standard list, it was necessary to determine which of the candidates was the most likely to be correct. Simple rules were used, removing gene mentions that referred to the same gene by different names. Otherwise, the best match was accepted. We believe that our results demonstrate that metric-based methods are insufficient, even when coupled with orthographic similarity between two tokens.

#### Team 14 (Patrick Ruch, University and Hospitals of Geneva)

For gene normalization, the GeneTeam system [9] used an automatic text categorization framework for large multiclass classification problems [49]. Unlike most automatic text categorization systems, which rely on data-intensive models extracted from large sets of training data, the GeneTeam categorizer is largely data-independent and a small sample is sufficient to tune the system. Approximately 3 person-days were used for experiments in the GN task of BioCreative II. The abstract, the title, and other fields of PubMed/MEDLINE records were used to generate the runs. Each article was augmented with its assigned Medical Subject Headings by querying PubMed/MEDLINE. Synonyms were handled as if they were different entities by the categorizer, but in the final output only top-ranked synonyms were returned.

In addition to the lexicon provided by the organizers, some synonyms were also added to this resource. The Categorizer was based on two ranking modules: a pattern matcher and a vector space retrieval engine [50]. For these experiments, the vector space retrieval engine used a slightly modified dtu.dtn formula (term frequency, document frequency, and pivoted normalization). The system produced a score computed as a linear combination between the retrieval status value of the retrieval engine, the maximal length of the matching category, and the number of matching features (Boolean scoring). The pattern matcher module used both stems and linguistically motivated indexing units, in particular noun phrases. A simple stemmer was used to handle plural English forms and a list of task-specific stop words was used, together with a list of stop categories. Stop words were removed before categorization, while stop categories were removed after categorization. These lists were established either manually using the tuning data, or automatically using differential frequency lists established on biomedical (PubMed/MEDLINE) and newspaper (Wall Street Journal) corpora. Using a data-driven argumentative classifier [49], we also attempted to augment the weight of particular sentences (purpose and conclusion) in the input abstract. The resulting output of the system produced a ranked list of categories.

To meet the requirements of the GN task, for every category the system attempted to recover the string that best represented the text form of the predicted category. This passage recovery was based on the computation of a string-to-string edit distance between the predicted category and the input text.

#### Team 58 (Chengjie Sun, Harbin Institute of Technology)

A Maximum Entropy binary classifier was used to distinguish correct from incorrect synonym matches, where good matches were positively labeled and bad matches negatively labeled [51]. To create training data for the classifier, every synonym in the lexicon provided by the organizers (entrezGeneLexicon.list file) was matched to each training document using a strict matching criterion (although a looser matching criterion might have been better). For each match, the system extracted the matching text plus the three words preceding and following the match and the normal form (unique identifier) causing the match. For the training data, if the normal form for a match was in the normalized gene list for that document, then the match was labeled positive; otherwise, it was labeled negative. This provided the large set of positive and negative matches required to train a Maximum Entropy classifier.

To classify a new abstract, the system first extracted all the synonym matches that occurred within it. Then, for each match, the classifier determined whether it was positive or negative. For each positive match, the identifier associated with the match was added to the document's normalized gene list.

## Additional data files

The following additional data are available with the online version of this paper. Additional data file 1 contains a glossary of terms. Additional data file 2 contains a table summarizing scores from all gene normalization runs. Additional data file 3 contains a table summarizing genes missed by all systems. Additional data file 4 contains a table listing of gene identifiers found by only one system.

## Abbreviations

EGID, Entrez Gene identifier; FN, false negatives; FP, false positives; GM, gene mention; GN, gene normalization; GO, Gene Ontology; GOA, Gene Ontology Annotation; HGNC, Hugo Gene Name Consortium; NCBI, National Center for Biotechnology Information; PPI, protein-protein interaction; SVM, support vector machine; TP, true positives; UMLS, Unified Medical Language System.

## Competing interests

The work of Xinglong Wang was funded by ITI Life Sciences, Scotland, whose mission is to explore commercialization of promising technologies in the life sciences. Funding for the work of Dr. Juliane Fluck was provided in part by the EU project @neurIST and in part by the Fraunhofer Institute (a public non-profit research organization), which also licenses the ProMiner software. The work of Rafael Torres was supported by Bioalma. The work of Martijn Schuemie was performed at the Biosemantics Group of the Erasmus University Medical Center in Rotterdam. This group was funded in part by Biorange project 4.1.1 of the Netherlands BioInformatics Center (NBIC), and has received license fees paid by the company Knewco for use of the Peregrine software.

The remaining authors: Alexander Morgan, Zhiyong Lu, Aaron Cohen, Patrick Ruch, Anna Divoli, Katrin Fundel, Robert Leaman, Jörg Hakenberg, Chenjie Sun, Heng-hui Liu, Michael Krauthammer, William Lau, Hongfang Liu, Chun-Nan Hsu, K. Bretonnel Cohen, and Lynette Hirschman, declare that they have no competing interests.

## Authors' contributions

The first author (AAM) developed the human gene normalization task, supervised the preparation of the data, developed the evaluation software and was responsible for preparing the evaluation results, including the results on the 'combined systems.' KBC contributed to the writing and to the background for gene normalization work. The corresponding author (LH) was responsible for overall project management, analysis, and the writing of the article. The remaining authors (ZL, XW, AMC, JF, RP, AD, KF, RL, JH, CS, HHL, RT, MK, WWL, HL, CNH, MS), are listed in order of their team identifiers; these authors were participants in the Gene Normalization challenge evaluation and provided the system summaries in the Methods section.

## Supplementary Material

Additional file 1 Click here for file

Additional file 2 Click here for file

Additional file 3 Click here for file

Additional file 4 Click here for file
