# Supplementary material for: Overview of BioCreative II gene normalization
Source: Genome Biol. 2008 Sep 1;9(Suppl 2):S3. doi: 10.1186/gb-2008-9-s2-s3 (PMC2559987; doi:10.1186/gb-2008-9-s2-s3)
Supplement: Additional file 1 [file gb-2008-9-s2-s3-s1.doc]

# Glossary

### Biological resources

BioThesaurus: a web-based system designed to map a comprehensive collection of protein and gene names to UniProt Knowledgebase (UniProtKB) protein entries (Liu et al., 2006a, 2006b). (http://pir.georgetown.edu/pirwww/iprolink/biothesaurus.shtml)

Brown Corpus: A carefully compiled selection of about 1 million words of American English drawn from a wide variety of sources. The corpus was developed by Francis and Kucera in 1964 and is now available at http://icame.uib.no/brown/bcm.html.

Entrez Gene: a searchable database of genes, from RefSeq genomes, and defined by sequence and/or located in the NCBI (http://www.ncbi.nlm.nih.gov/sites/entrez?db=gene).

GeneRIF (Gene Reference Into Function): a short (255 characters or less) statement about the function of a gene associated with a specific entry in the Entrez Gene database. (http://www.ncbi.nlm.nih.gov/projects/GeneRIF/)

GO (Gene Ontology): GO supports comparative genomics through the development of three structured controlled vocabularies (ontologies) that describe gene products in terms of their associated biological processes, cellular components and molecular functions in a species-independent manner. (http://www.geneontology.org/)

GOA (Gene Ontology (GO) Annotation): provides high-quality Gene Ontology (GO) annotations to proteins in the UniProt Knowledgebase (UniProtKB) and International Protein Index (IPI). (http://www.ebi.ac.uk/GOA/)

GoPubMed: a knowledge-based search engine for biomedical texts; it uses the Gene Ontology as to structure the contents of the MEDLINE data base. (http://www.gopubmed.org/)

HGNC (HUGO Gene Nomenclature Committee): The organizational that provides official gene names and gene symbols (a short-form abbreviation) for the human genome. (http://www.genenames.org/)

HUGO (Human Gene Organization): the international organization of scientists involved in human genetics; it is responsible for HGNC or HUGO Gene Nomenclature Committee. (http://www.hugo-international.org/)

KIAA: A project of the Kazusa cDNA project focused particularly on large cDNAs encoding large proteins. more than 2000 human genes, referred to as ‘KIAA’ genes, were initially identified through this cDNA project. (http://www.kazusa.or.jp/huge/)

MeSH (Medical Subject Headings): the National Library of Medicine’s controlled vocabulary thesaurus consisting of sets of terms naming descriptors in a hierarchical structure that permits searching at various levels of specificity. (http://www.nlm.nih.gov/mesh/)

OMIM (Online Mendelian Inheritance in Man): a catalog of human genes and genetic disorders containing textual information and references, as well as links to MEDLINE and sequence records in the Entrez system. (http://www.ncbi.nlm.nih.gov/sites/entrez?db=omim)

PIR (Protein Information Resource): an integrated public bioinformatics resource to support genomic and proteomic research, and scientific studies. (http://pir.georgetown.edu/)

RefSeq: The Reference Sequence (RefSeq) collection aims to provide a comprehensive, integrated, non-redundant set of sequences, including genomic DNA, transcript (RNA), and protein products. (http://www.ncbi.nlm.nih.gov/RefSeq/)

Swiss-Prot: a manually curated biological database of protein sequences. http://en.wikipedia.org/wiki/SwissProt. (http://au.expasy.org/sprot/)

TrEMBL (Translated EMBL) is a very large protein database in Swiss-Prot format generated by computer translation of the genetic information from the EMBL Nucleotide Sequence Database. (http://www.ebi.ac.uk/trembl/).

UMLS (Unified Medical Language System): resources and associated software tools distributed by NLM for use by system developers for use in information systems that create, process, retrieve, integrate, and/or aggregate biomedical and health data and information, as well as in informatics research; the resources include the Metathesaurus®, the Semantic Network, and the SPECIALIST Lexicon. They are distributed with flexible lexical tools and the MetamorphoSys install and customization program. (http://www.nlm.nih.gov/research/umls/)

UniProt: the universal protein database, a central repository of protein data created by combining Swiss-Prot, TrEMBL and PIR. This makes it the world's most comprehensive resource on protein information. (http://www.pir.uniprot.org/)

### Natural language/text mining terms

AdaBoost (Adaptive Boosting): a machine learning meta-algorithm used in conjunction with other learning algorithms to improve their performance.

Ambiguity: a word is said to be “ambiguous” if it can be interpreted in more than one way. For example, the gene term “clk” is ambiguous in Drosophila, because it has been used to the “clock” gene and the “period” gene.

Bag of words: a model that assumes a document is represented as an unordered collection of words, disregarding word order.

Cosine similarity: a measure used to compare two vectors using the cosine of the angle between the two vectors; for text mining, the two vectors are often the normalized number of occurrences of non-stop words, where each dimension of the vector corresponds to a separate term.

CRF (Conditional Random Field): a probabilistic framework for labeling and segmenting structured data, such as sequences, based on defining a conditional probability distribution over label sequences given a particular observation sequence, rather than a joint distribution over both label and observation sequences, as done for Hidden Markov Models (HMMs).

Dice Coefficient: a similarity measure related to the Jaccard index (size of intersection divided by the size of the union of the sample sets); as a string similarity measure, the coefficient may be calculated for two strings, *x* and *y* using bigrams.

Edit distance: the number of operations required to transform one string into another string; algorithms used to calculate this metric include, among others, Hamming distance, Levenshtein distance and Jaro-Winkler distance.

Enumeration: the listing of multiple terms, often in a compact form that requires expansion into full form for further processing, as in: *"protein kinase C isoforms alpha, epsilon, and zeta"* corresponding to the full forms “*protein kinase C isoforms alpha”, “protein epsilon”,* and *“protein kinase C isoforms zeta”.*

HMM (Hidden Markov Model): a statistical model where the system being modeled is assumed to be a Markov process with unknown parameters, and the challenge is to determine the hidden parameters from the observable parameters. HMMs have been used extensively for pattern recognition in sequences, such as speech, text, and bioinformatics.

Jaro-Winkler: The Jaro string similarity measure is based on the number and

order of characters that are common to two strings. A variant of the Jaro measure due to Winkler rewards strings which have a common prefix

Lexicon: A linguistic resource that lists words and associated information for each word, such as variant forms (synonyms), the root or canonical form, part of speech, etc. As used here: a list of words (e.g., gene or protein names) with their unique database identifier.

Morphology: the study of how words are constructed in language from meaningful subparts or morphemes, e.g., ‘endonuclease” consists of the root ‘nucleus’, the prefix ‘endo-‘ and the suffix ‘-ase’.

NER (Named-entity recognition): the labeling of mentions of names in free text, generally according to a limited set semantic classes, e.g., person, organization and location names; or gene names and protein names for biology.

Part of speech: a set of grammatical tags used to label the words of a language, e.g., noun, verb, preposition.

Soft TF/IDF: an augmentation of TF-IDF with approximate token matching using a secondary string similarity measure, such as Jaro-Winkler.

Stemming: the process of reducing a word to its simplest form for efficient searching by removing prefixes and suffixes (endings), such as (in English) -s, -ed, and -ing.

Stop words: A word which is ignored in a query because it is so common that it does not contribute to the search; typical stop words in English are “the”, “a”, “of” etc.

SVM (Support Vector Machine): a set of related supervised learning methods used for classification and regression; an SVM performs classification by constructing an *N*-dimensional hyperplane that optimally separates the data into two categories.

Synonym: a word that can be interchanged with another word in a context; in genomics, for example, a gene name and its gene symbol would be considered to be synonyms.

Syntax: the study of the rules that govern sentence formation by the combination of lexical items into phrases, including, for English, word order and inflection – the addition of suffixes such as tense markers (-ed) or agreement (-s).

Tagger: a program that labels occurrences of words or phrases in a document according to membership in a class; for example, a part of speech tagger labels words in a document by their part of speech; a gene name tagger labels words or sequences of phrases as gene names.

Text string: a sequence of characters in text.

TF/IDF (Term Frequency/Inverse Document Frequency): a weight used to evaluate how important a word is to a document in a collection or corpus, where term frequency is the frequency of a term within a given document and inverse document frequency is the inverse frequency of the term within the larger document collection or corpus.

Tokenization: the process of dividing a sequence of symbols into elements (typically, ‘words’ or lexemes) for further processing.

### Natural language tools or systems:

AbGene: a downloadable gene and protein name mention tagger (ftp://ftp.ncbi.nlm.nih.gov/pub/tanabe)

ABNER: an open source tool for automatically tagging genes, proteins and other entity names in text (http://www.cs.wisc.edu/~bsettles/abner)

KeX: an open source protein name mention tagger (http://www.hgc.jp/service/tooldoc/KeX)

LingPipe: a downloadable set of tools for information extraction and data mining, including part of speech tagging, named entity recognition, entity tracking, chunking, and text classification (http://www.alias-i.com/lingpipe/)

NLPBA (Natural Language Processing in Biomedicine and its Applications): a workshop held at COLING 2004, focused on evaluation of biological named entity tagging.
