# Supplementary material for: Overview of BioCreative II gene normalization
Source: Genome Biol. 2008 Sep 1;9(Suppl 2):S3. doi: 10.1186/gb-2008-9-s2-s3 (PMC2559987; doi:10.1186/gb-2008-9-s2-s3)
Supplement: Additional file 2 [file gb-2008-9-s2-s3-s2.doc]

## Scores from all gene normalization runs

|  | Micro-Average | | | | | | Macro-Average | | |
| --- | --- | --- | --- | --- | --- | --- | --- | --- | --- |
| Team_Run | Recall | Precision | F-measure | True Positive | False Positive | False Negative | Recall | Precision | F-measure |
| Maximum | 0.875 | 0.841 | 0.810 | 687 | 840 | 541 | 0.876 | 0.898 | 0.811 |
| Top Quartile | 0.767 | 0.782 | 0.770 | 602 | 271 | 282 | 0.817 | 0.807 | 0.754 |
| Median | 0.750 | 0.797 | 0.773 | 582 | 149 | 227 | 0.832 | 0.784 | 0.759 |
| 3rd Quartile | 0.732 | 0.735 | 0.717 | 575 | 198 | 211 | 0.775 | 0.773 | 0.700 |
| Minimum | 0.311 | 0.361 | 0.370 | 244 | 102 | 98 | 0.474 | 0.316 | 0.342 |
| T004_1 | 0.734 | 0.841 | 0.784 | 576 | 109 | 209 | 0.876 | 0.776 | 0.775 |
| T004_2 | 0.743 | 0.829 | 0.784 | 583 | 120 | 202 | 0.865 | 0.784 | 0.777 |
| T004_3 | 0.748 | 0.820 | 0.782 | 587 | 129 | 198 | 0.854 | 0.787 | 0.772 |
| T006_1 | 0.601 | 0.767 | 0.674 | 472 | 143 | 313 | 0.794 | 0.700 | 0.686 |
| T006_2 | 0.606 | 0.767 | 0.677 | 476 | 145 | 309 | 0.787 | 0.705 | 0.685 |
| T006_3 | 0.782 | 0.597 | 0.677 | 614 | 414 | 171 | 0.629 | 0.808 | 0.666 |
| T007_1 | 0.707 | 0.731 | 0.719 | 555 | 204 | 230 | 0.755 | 0.728 | 0.687 |
| T007_2 | 0.703 | 0.746 | 0.724 | 552 | 188 | 233 | 0.770 | 0.725 | 0.694 |
| T007_3 | 0.699 | 0.749 | 0.723 | 549 | 184 | 236 | 0.770 | 0.717 | 0.688 |
| T013_1 | 0.768 | 0.833 | 0.799 | 603 | 121 | 182 | 0.848 | 0.803 | 0.779 |
| T013_2 | 0.730 | 0.835 | 0.779 | 573 | 113 | 212 | 0.856 | 0.770 | 0.749 |
| T013_3 | 0.803 | 0.779 | 0.790 | 630 | 179 | 155 | 0.816 | 0.829 | 0.773 |
| T014_1 | 0.485 | 0.762 | 0.593 | 381 | 119 | 404 | 0.783 | 0.575 | 0.584 |
| T014_2 | 0.483 | 0.471 | 0.477 | 379 | 425 | 406 | 0.474 | 0.517 | 0.419 |
| T014_3 | 0.655 | 0.479 | 0.553 | 514 | 559 | 271 | 0.543 | 0.701 | 0.555 |
| T017_1 | 0.708 | 0.720 | 0.714 | 556 | 216 | 229 | 0.764 | 0.754 | 0.709 |
| T017_2 | 0.641 | 0.806 | 0.714 | 503 | 121 | 282 | 0.845 | 0.701 | 0.710 |
| T017_3 | 0.757 | 0.631 | 0.688 | 594 | 348 | 191 | 0.671 | 0.804 | 0.688 |
| T030_1 | 0.661 | 0.716 | 0.687 | 519 | 206 | 266 | 0.736 | 0.695 | 0.649 |
| T030_2 | 0.666 | 0.702 | 0.684 | 523 | 222 | 262 | 0.729 | 0.698 | 0.645 |
| T030_3 | 0.707 | 0.580 | 0.637 | 555 | 402 | 230 | 0.616 | 0.737 | 0.617 |
| T034_1 | 0.815 | 0.792 | 0.804 | 640 | 168 | 145 | 0.815 | 0.841 | 0.782 |
| T034_2 | 0.847 | 0.723 | 0.780 | 665 | 255 | 120 | 0.736 | 0.870 | 0.758 |
| T034_3 | 0.789 | 0.739 | 0.763 | 619 | 219 | 166 | 0.754 | 0.821 | 0.740 |
| T036_1 | 0.713 | 0.520 | 0.602 | 560 | 516 | 225 | 0.562 | 0.764 | 0.595 |
| T042_1 | 0.833 | 0.789 | 0.810 | 654 | 175 | 131 | 0.836 | 0.866 | 0.811 |
| T042_2 | 0.875 | 0.496 | 0.633 | 687 | 699 | 98 | 0.567 | 0.898 | 0.649 |
| T042_3 | 0.725 | 0.707 | 0.716 | 569 | 236 | 216 | 0.732 | 0.760 | 0.706 |
| T058_1 | 0.429 | 0.361 | 0.392 | 337 | 596 | 448 | 0.570 | 0.476 | 0.382 |
| T058_2 | 0.415 | 0.375 | 0.394 | 326 | 543 | 459 | 0.611 | 0.475 | 0.398 |
| T058_3 | 0.331 | 0.419 | 0.370 | 260 | 361 | 525 | 0.671 | 0.371 | 0.342 |
| T101_1 | 0.762 | 0.751 | 0.756 | 598 | 198 | 187 | 0.771 | 0.808 | 0.741 |
| T101_2 | 0.743 | 0.801 | 0.771 | 583 | 145 | 202 | 0.820 | 0.789 | 0.755 |
| T101_3 | 0.734 | 0.804 | 0.767 | 576 | 140 | 209 | 0.820 | 0.779 | 0.749 |
| T102_1 | 0.415 | 0.585 | 0.486 | 326 | 231 | 459 | 0.660 | 0.420 | 0.431 |
| T102_2 | 0.521 | 0.552 | 0.536 | 409 | 332 | 376 | 0.619 | 0.535 | 0.494 |
| T102_3 | 0.790 | 0.425 | 0.552 | 620 | 840 | 165 | 0.483 | 0.814 | 0.559 |
| T104_1 | 0.743 | 0.807 | 0.774 | 583 | 139 | 202 | 0.840 | 0.785 | 0.773 |
| T104_2 | 0.758 | 0.779 | 0.768 | 595 | 169 | 190 | 0.804 | 0.803 | 0.763 |
| T107_1 | 0.740 | 0.784 | 0.761 | 581 | 160 | 204 | 0.818 | 0.776 | 0.739 |
| T108_1 | 0.796 | 0.655 | 0.719 | 625 | 329 | 160 | 0.685 | 0.826 | 0.708 |
| T108_2 | 0.782 | 0.690 | 0.733 | 614 | 276 | 171 | 0.723 | 0.814 | 0.720 |
| T108_3 | 0.749 | 0.726 | 0.737 | 588 | 222 | 197 | 0.761 | 0.785 | 0.724 |
| T109_1 | 0.824 | 0.743 | 0.781 | 647 | 224 | 138 | 0.780 | 0.848 | 0.775 |
| T109_2 | 0.792 | 0.764 | 0.778 | 622 | 192 | 163 | 0.806 | 0.815 | 0.767 |
| T109_3 | 0.769 | 0.790 | 0.779 | 604 | 161 | 181 | 0.817 | 0.806 | 0.764 |
| T110_1 | 0.629 | 0.783 | 0.698 | 494 | 137 | 291 | 0.830 | 0.691 | 0.685 |
| T110_2 | 0.641 | 0.738 | 0.686 | 503 | 179 | 282 | 0.794 | 0.708 | 0.674 |
| T110_3 | 0.622 | 0.732 | 0.672 | 488 | 179 | 297 | 0.785 | 0.698 | 0.669 |
| T111_1 | 0.327 | 0.652 | 0.436 | 257 | 137 | 528 | 0.790 | 0.331 | 0.362 |
| T111_2 | 0.311 | 0.705 | 0.431 | 244 | 102 | 541 | 0.828 | 0.316 | 0.357 |
| T111_3 | 0.664 | 0.717 | 0.689 | 521 | 206 | 264 | 0.731 | 0.706 | 0.664 |
| T113_1 | 0.745 | 0.723 | 0.734 | 585 | 224 | 200 | 0.779 | 0.795 | 0.733 |
| T113_2 | 0.761 | 0.752 | 0.756 | 597 | 197 | 188 | 0.782 | 0.810 | 0.745 |
