# Supplementary material for: Overview of BioCreative II gene normalization
Source: Genome Biol. 2008 Sep 1;9(Suppl 2):S3. doi: 10.1186/gb-2008-9-s2-s3 (PMC2559987; doi:10.1186/gb-2008-9-s2-s3)
Supplement: Additional file 3 [file gb-2008-9-s2-s3-s3.doc]

## Genes missed by all systems

The table shows PMID, Entrez Gene ID, text snippet from Gold Standard, lexical entry from Entrez Gene, and diagnosis of problem.

| **PMID** | **EG ID** | **Text in Gold Standard** | **Synonym in Gold Std** | **Diagnosis** |
| --- | --- | --- | --- | --- |
| 8706133 | 6902 | cofactors A | beta-tubulin cofactor A | Missing context |
| 9543345 | 4513 | COX subunit II | COX2 | Missing synonym |
| 9618483 | 239 | 12S-lipoxygenase | arachidonate 12-lipoxygenase | Missing synonym |
| 9748262 | 4824 | NK-3 | NK3 Homeobox or NKX3-1 | Ambiguity |
| 9892355 | 2566 | gamma2 subunit of GABA(A) receptors | gamma-aminobutyric acid (GABA) A receptor, gamma 2 | Complex description |
| 9892355 | 81631 | Light chain-3 of microtubule- associated proteins 1A and 1B | Microtubule-associated proteins 1A/1B light chain 3B precursor | Complex description |
| 9892355 | 84557 | light chain-3 of microtubule-associated proteins 1A | microtubule-associated proteins 1A/1B light chain 3 | Complex description |
| 9932288 | 348932 | XT2 | XTRP2 | Missing synonym, ambiguity |
| 10235267 | 5970 | p65 subunit of NF-kappaB | NFKB | Complex description |
| 10458166 | 4090 | SMADs 1, 5 | SMAD 5 | Conjunction |
| 10458166 | 4093 | SMADs 1, 5 and 8 | SMAD8A, SMAD8B | Missing synonym |
| 10531035 | 7325 | Ubc4 | UbcH8 | Missing synonym, ambiguity |
| 10766764 | 3779 | beta1 | potassium large conductance calcium-activated channel, subfamily M, beta member 1 | Missing context |
| 10766764 | 10242 | beta2 | ...beta member 2 | Missing context |
| 10766764 | 27094 | beta3a-c | ...beta member 3 | Missing context |
| 11003675 | 9662 | Protein 4.1 R-135 | centrosomal protein 135kDa | Missing synonym |
| 11311562 | 3313 | Mt-Hsp70 | heat shock 70kD protein 9B (mortalin-2) | Missing synonym |
| 11559747 | 5252 | Polycomblike | PHD finger protein 1 | Missing synonym |
| 12027893 | 3643 | insulin- and EGF-receptor | insulin receptor | Conjunction |
| 12093742 | 2188 | FA proteins A, C, G and F | Fanconi anemia, complementation group F | Missing synonym |
| 12093742 | 2189 | FA proteins A, C, G | Fanconi anemia group G protein | Missing synonym |
| 14626429 | 5925 | pRb | retinoblastoma 1 (including osteosarcoma) | Missing synonym, ambiguity |
