# Supplementary material for: Overview of BioCreative II gene normalization
Source: Genome Biol. 2008 Sep 1;9(Suppl 2):S3. doi: 10.1186/gb-2008-9-s2-s3 (PMC2559987; doi:10.1186/gb-2008-9-s2-s3)
Supplement: Additional file 4 [file gb-2008-9-s2-s3-s4.doc]

## Listing of gene identifiers found by only one system

Table includes PubMedID, Entrez Gene ID, the snippet of text provided by the expert annotator, presence or absence of a conjunction, the team that provided the correct identifier, and its rank (based on micro-averaged F-measure).

|  | PMID | EntrezGene ID | Text from Gold Standard | Conjunction | System | Rank |
| --- | --- | --- | --- | --- | --- | --- |
| 1 | 8706133 | 6903 | cofactors A, D, E, and C | x | T036_1 | 17 |
| 2 | 8706133 | 6904 | cofactors A, D | x | T109_1 | 5 |
| 3 | 9647693 | 3064 | huntingtin |  | T034_1 | 2 |
| 4 | 10235267 | 3054 | VP16 |  | T036_1 | 17 |
| 5 | 10458166 | 4088 | SMADs 2 and 3 | x | T109_1 | 5 |
| 6 | 10607840 | 29989 | hOBP (IIb) |  | T006_2 | 16 |
| 7 | 10954742 | 3588 | beta chain of the IL-10 receptor |  | T013_1 | 3 |
| 8 | 11196645 | 2770 | Gi |  | T110_1 | 13 |
| 9 | 11698403 | 8790 | GDP-fucose pyrophosphorylase |  | T101_2 | 7 |
| 10 | 1897791 | 79751 | mitochondrial glutamate transporter |  | T111_3 | 14 |
| 11 | 11909966 | 3066 | HDACs 1 and 2 | x | T102_3 | 19 |
| 12 | 12862317 | 51154 | acidic ribosomal protein PO |  | T017_1 | 12 |
